# Infections of Leisure, Fifth Edition

**DOI:** 10.3201/eid2509.190634

**Published:** 2019-09

**Authors:** Katie Fullerton

**Affiliations:** Centers for Disease Control and Prevention, Atlanta, Georgia, USA

**Keywords:** leisure activities, infection, book review, tattoos, piercings, food, pets, zoonoses, bacteria

The fifth edition of *Infections of Leisure* is a comprehensive, detailed survey of infectious hazards associated with a number of activities we may do in our leisure time, such as eating exotic cuisines, challenging ourselves at high altitude, owning pets, playing at the seashore or in a pool, getting tattoos or body piercings, and traveling abroad (Figure). This edition has been updated to “… incorporate new and changing pathogens that can compromise our leisure activities” (*1*).

**Figure:**
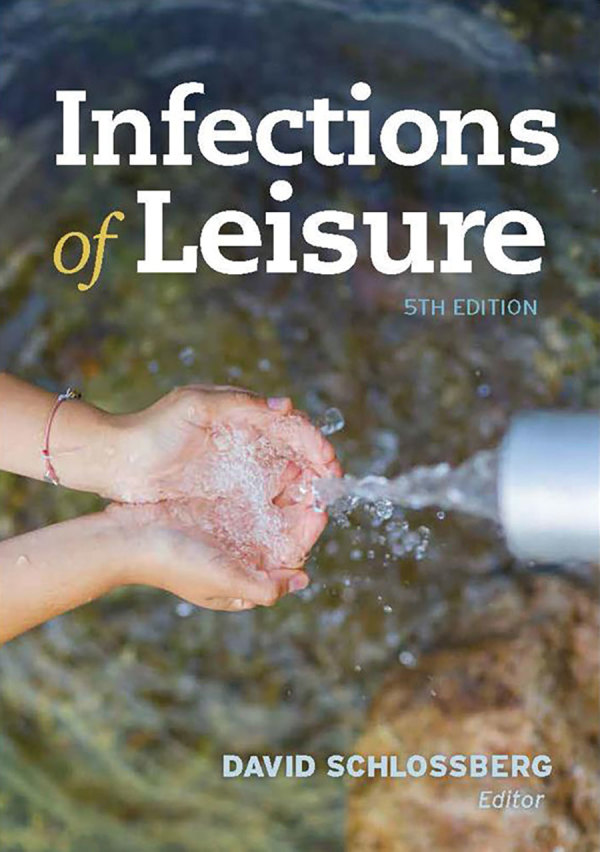
Infections of Leisure, Fifth Edition

As in previous editions, this edition covers infections associated with just about any type of leisure activity one can imagine and contains good doses of history alongside clinical, microbiological, and epidemiologic information. I was fascinated by the history of humankind’s attempts to eradicate rats (Chapter 9: Diseases Transmitted by Man’s Worst Friend: The Rat), a notable failed effort; the anecdote about the Duke of Richmond, the Governor General of British North America (now Canada) in 1817, who died from rabies after a fox bite that occurred while he was trying to separate his hunting dogs from a fox (Chapter 11: The Ancient Curse: Rabies); and the history of tattooing, dating back to the Egyptians (Chapter 15: Infections from Body Piercing and Tattoos). The chapters covering zoonoses from domestic pets (dogs, cats, birds, and other less common house pets) are comprehensive, covering bacterial zoonoses that are topical, such as campylobacteriosis associated with exposure to puppies (*2*), as well as quantifying the number of ferret-owning households in the United States (334,000).

Of note, the next edition could expand a bit on harmful algal bloom–associated illnesses, which were only briefly mentioned in the fifth edition as cyanobacterium infections in the chapter on fresh water. These illnesses have been reported in conjunction with untreated recreational water and drinking water and can encompass more than cyanobacterial toxins (*3*). *Shigella*, a bacterium that causes travelers’ diarrhea and foodborne gastrointestinal disease, is more frequently being reported in association with sexual activity among men who have sex with men and could potentially be included in the chapter on sexually transmitted diseases, as could other enteric infections that are transmitted person-to-person through the fecal-oral route (*4*).

The index proves most useful for locating information that might be associated with multiple leisure activities; leptospirosis, for example, in addition to being discussed at length in the section on rat-associated infections, is also discussed in the chapters on garden-associated infections, dog-associated infections, international travel–associated infections, and infections at high altitude. The breadth of infections covered in this book is extensive, from amnesiac shellfish poisoning to Carrion disease (causative agent *Bartonella bacilliformis*), monkeypox, and weeverfish envenomation.

This book is readable, topical, and a useful resource for those who want to balance the relaxation or excitement they may derive from their chosen leisure pursuits with an in-depth knowledge of all the things that could go wrong. Of note, a section in each chapter focuses on prevention measures that can be taken. This book is appropriate for undergraduate students; professionals; and clinical, microbiology, and public health practitioners.
